# Prevalence of diabetes in Brazil over time: a systematic review with meta-analysis

**DOI:** 10.1186/s13098-016-0181-1

**Published:** 2016-09-07

**Authors:** Gabriela H. Telo, Felipe Vogt Cureau, Martina S. de Souza, Thais S. Andrade, Fabiana Copês, Beatriz D. Schaan

**Affiliations:** 1 Internal Medicine Department, Medical School, Universidade Federal do Rio Grande do Sul, Rua Ramiro Barcelos, 2350, Porto Alegre, CEP 90035-903 Brazil; 2 Postgraduate Program in Endocrinology, Universidade Federal do Rio Grande do Sul, Porto Alegre, Brazil; 3 Departament of Nutrition, Universidade Federal do Pampa, Campus Itaqui, Brazil; 4 Endocrine Division, Hospital de Clínicas de Porto Alegre, Porto Alegre, Brazil

**Keywords:** Type 2 diabetes, Prevalence, Brazil

## Abstract

Diabetes is one of the most important epidemic diseases of this century and the number of people with diabetes has more than doubled over the past three decades. Our aim was to estimate the prevalence of diabetes in the adult Brazilian population and analyze the trends for the last three decades through a systematic review with meta-analysis. This review included observational studies published between 1980 and 2015, which were independently identified by two reviewers in five databases. Random effect models were used to estimate the prevalence and trends of diabetes. In total, 50 articles were included in this review. Three different patterns for diabetes diagnosis were identified: self-report (36 studies), fasting glucose (7 studies), and complex diagnosis (fasting glucose, oral glucose tolerance test, and self-report; 7 studies). The prevalence of diabetes was 5.6 % (95 % CI 5.0–6.3; I^2^ = 100 %) by self-report, 6.6 % (95 % CI 4.8–8.9; I^2^ = 94 %) by fasting glucose, and 11.9 % (95 % CI 7.7–17.8 I^2^ = 100 %) by complex diagnosis. In trend analyses, we observed an increase in the prevalence of diabetes over time. The biggest increase was detected in studies using complex diagnosis: 7.4 % (95 % CI 7.1–7.7) in the 1980s to 15.7 % (95 % CI 9.8–24.3) in the 2010s. In conclusion, despite high heterogeneity, this study observed a high prevalence of diabetes in Brazilian adults over time and with a progressive increase in the last 35 years.

**Electronic supplementary material:**

The online version of this article (doi:10.1186/s13098-016-0181-1) contains supplementary material, which is available to authorized users.

## Background

Diabetes is one of the most important epidemic diseases of this century. The global prevalence of type 2 diabetes is increasing worldwide as a result of population ageing [1], rising rates of overweight and obesity in adults as well as youth [2], and reduced risk of mortality among patients with diabetes [3]. The number of people with diabetes has more than doubled over the past three decades in nearly every nation of the world [4]. In 2014, the global prevalence of diabetes was estimated to be 9 % among adults older than 18 years old [5]. It has been projected that there will be 366 million adults with diabetes in 2030 [6] and, despite recent declines in mortality, diabetes will be the seventh leading cause of death in 15 years [7], making it one of the most important public health challenges to all nations [8].

The major burden of diabetes is now taking place in developing rather than in developed countries, and 80 % of patients with diabetes live in less developed areas [8]. All the Latin American countries have undergone rapid demographic, epidemiological and nutritional transitions [9], which strongly contributed to the increasing prevalence of diabetes. Brazil is one of the most important examples of this alarming problem in less developed societies with the fourth largest number of people with type 2 diabetes [10]. In the South and Central America region, 8.0–11.3 % of the adult population have diabetes. Of these, 39.0 % are undiagnosed. Moreover, Brazil has the highest number of people with diabetes in the region. In 2015, in Latin America, almost 250,000 adults died as a result of diabetes, of which half of the deaths occurred in Brazil [11]. The International Diabetes Federation estimated the prevalence of diabetes in Brazil to be 10.3 % in 2012 [12], which represents a gradual increase over the past three decades.

This rising diabetes prevalence has translated into a 60 % increase in the attributable risk ratio for cardiovascular diseases associated with diabetes [13]. However, there is a lack of nationwide prevalence data over time in most emerging countries. In Brazil, although several cross-sectional analyses have been conducted to identify the prevalence of diabetes [9], no strong and consistent data is available to evaluate the trends over time. In this study, based on the hypothesis of increasing diabetes prevalence over decades and potential regional differences, we sought to investigate existing data sources on the prevalence of diabetes in Brazil and estimate the prevalence trends of diabetes for the last three decades in the adult Brazilian population through a systematic review with meta-analysis of observational studies.

## Methods

### Search strategy

A comprehensive literature search was conducted to identify articles containing information on diabetes prevalence in Brazil. Two reviewers independently searched in five different databases (PubMed, Cochrane Library, EMBASE, LILACS and SciELO). Search strategies were tested to find the appropriate medical subject heading terms for “Diabetes Mellitus”, “Brazil” and its regions, and “Prevalence”. The complete PubMed literature search strategy is described in the Additional file 1: Table S1. No language or age restrictions were applied. A manual search of the references of review articles, key publications, and abstracts from the two past years of the main national related meetings was also performed. All potentially eligible studies were considered for review. Duplicate data were excluded. The software EndNote version X6 (Thomson Reuters, New York, NY) was used for references selection management.

### Study selection

Additional file 2: Figure S1 shows the flow diagram of the studies included in the meta-analysis. Two independent and previously trained investigators performed the first titles and abstracts screening. All the selected studies were retrieved for full-text evaluation. We included all publications providing information on type 2 diabetes and our aimed end-point: prevalence, based on population-based cross-sectional and baseline of cohort studies among participants aged 18 years or older published between January 1980 and December 2015. Studies in which the sampling was not random or included less than 300 persons were excluded. Studies that assessed only specific subgroups not representative of its geographical strata were considered ineligible. Studies that included women or men only were considered eligible and were included only for analyses by gender. A third investigator solved disagreements between reviewers.

### Data extraction and assessment of study quality

Two reviewers separately evaluated the selected studies for data capture. The data were entered in a pretested Microsoft Office Excel™ spreadsheet based on the Strengthening in Epidemiology Statement (STROBE) checklist [14, 15]. The absolute rather than relative value of each variable was obtained. Any discordance between the data extracted was discussed until consensus was reached.

All studies were addressed for their capability to appropriately respond to our research question, as well as for selection, measurement, and analysis biases. Selection biases were defined by 20 % or more of refuses to participate in the study and by studies that used telephone calls as the only method for patient selection and interviews. Measurement bias was characterized based on diabetes diagnosis criteria: self-reported or measured. Analysis bias was defined as when a study did not consider the design effect to estimate the diabetes prevalence. Data regarding how the study handled missing data were also obtained. Sensitivity analyses were performed as pre-established to deal with potential study biases.

### Statistical analysis

Random effect models were used to calculate all point estimates and their 95 % confidence interval (95 % CI), as well as to estimate the prevalence of diabetes for the general population. Sensitivity analyses were performed by sex, decades, macro-region, and diagnostic criteria. Logit transformation was used to handle distribution asymmetry related to different prevalence measures. Continuity correction was used for adjustment when a discrete distribution was approximated by a continuous distribution. Prevalence was weighted by the inverse variance of logit. Pooled values were then converted to prevalence. Chi square was used to determine differences in prevalence rates among different decades. The Cochran Chi square and *I*^*2*^ test were used to evaluate statistical heterogeneity and consistency among the studies, and a value of *p* = 0.10 was used for significance.

Statistical analyses were performed using Stata version 12.1 (StataCorp LP, College Station, TX). MetaXL (EpiGear International, Sunrise Beach, Australia), an Excel comprehensive program for meta-analysis was used to build forest plots. Before start any study procedure, this study was registered [16] at PROSPERO, an international database of prospectively registered systematic reviews in health and social care, under the registration number of CRD42014010602.

## Results

### Synthesis of data

**Fig. 1:**
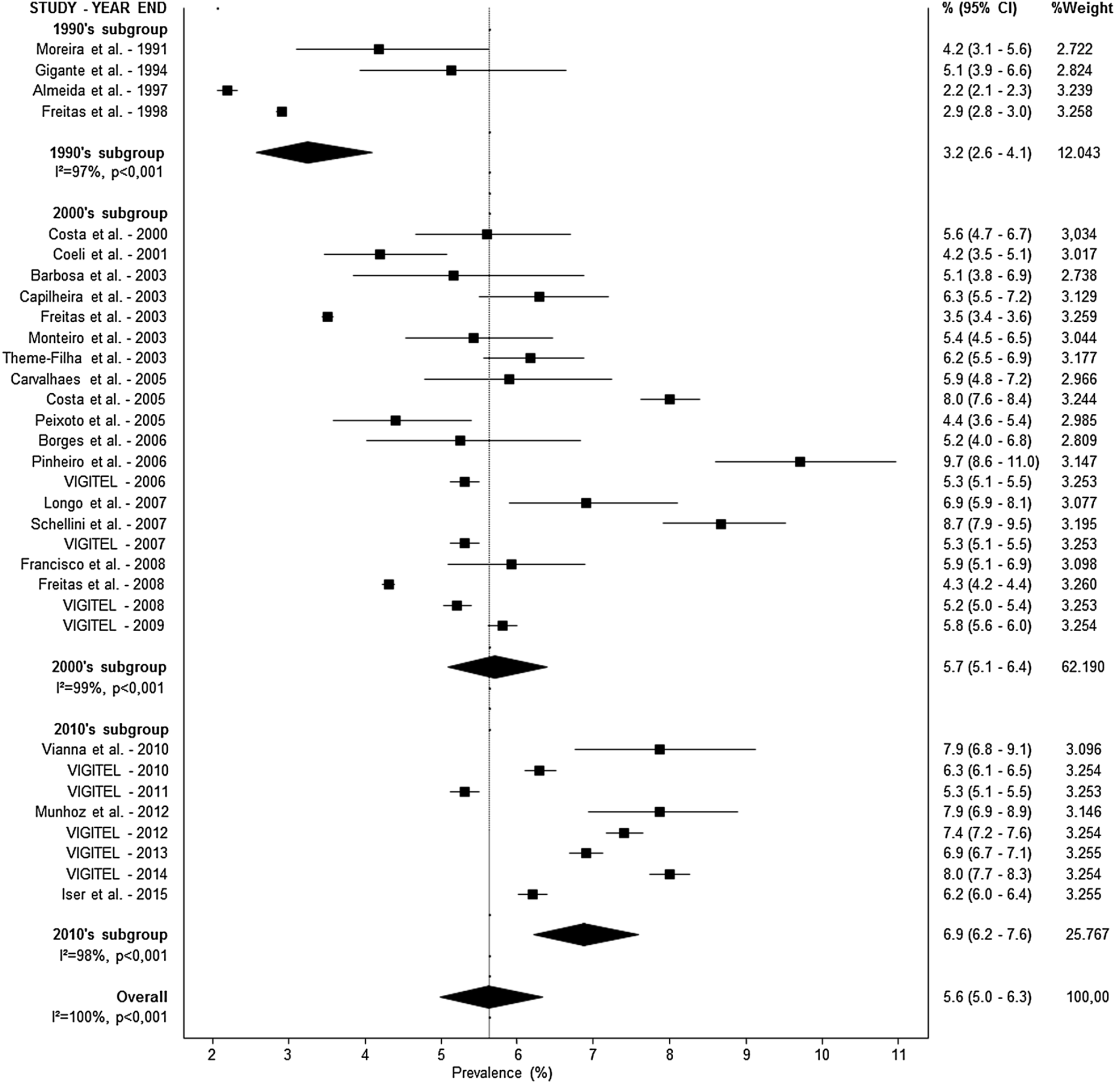
Forest plot representing diabetes prevalence rates by self-reported diagnosis and decades

The search retrieved 2522 articles from January 1980 to December 2015, of which 496 were duplicates and were excluded. Additional 1909 articles were removed based on title and abstracts; 117 full-text articles were assessed for eligibility, of which 40 met all the inclusion criteria. Manual search retrieved other ten articles, totalizing 50 studies (1,393,637 individuals) that were included in the final analyses. The flowchart of studies selection is presented in Fig. 1. Three different patterns for diabetes diagnosis were identified: self-report, fasting glucose, and complex diagnosis [e.g. fasting glucose + oral glucose tolerance test (OGTT) + self-report]. The characteristics of the included studies by diagnosis criteria and method of assessment are described in Table 1.

**Table 1:** Characteristics of the included studies by type of diagnosis

Study	Final year of data collection	Type of study	Sample size	Women (%)	Age criterion for study entry (years)	City, state, or region	Self-reported
Self-reported diagnosis							
Almeida et al. [17]	1997	Cross-sectional	20,287	45.7	30–69	Juiz de Fora, RJ	Personally
Barbosa et al. [18]	2003	Cross-sectional	835	59.7	>18	São Luiz, MA	Personally
Borges et al. [19]	2006	Cross-sectional	972	57.0	20–69	Pelotas, RS	Personally
Capilheira et al. [20]	2003	Cross-sectional	3100	56.6	>20	Pelotas, RS	Personally
Carvalhaes et al. [21]	2005	Cross-sectional	1410	61.4	>18	Botucatu, SP	By phone
Coeli et al. [22]	2001	Cross-sectional	2506	58.8	>30	Rio de Janeiro, RJ	Personally
Correia et al. [23]	2007	Cross-sectional	6431	100.0	20–49	Ceará	Personally
Costa et al. [24]	2005	Cross-sectional	19,252	57.1	>25	Five regions: North, Northeast, Midwest, Southeast, South	Personally
Costa et al. [25]	2000	Cross-sectional	1968	–	20–69	Pelotas, RS	Personally
Francisco et al. [26]	2008	Cross-sectional	2636	56.7	>18	Campinas, SP	Personally
Freitas et al. [27]	1998	Cross-sectional	217,709	–	>18	Five regions: North, Northeast, Midwest, Southeast, South	Personally
Freitas et al. [27]	2003	Cross-sectional	254,870	–	>18	Five regions: North, Northeast, Midwest, Southeast, South	Personally
Freitas et al. [27]	2008	Cross-sectional	271,677	–	>18	Five regions: North, Northeast, Midwest, Southeast, South	Personally
Fuchs et al. [28]	2005	Cross-sectional	1007	100.0	>18	Porto Alegre, RS	Personally
Gigante et al. [29]	1994	Cross-sectional	1035	56.0	>20	Pelotas, RS	Personally
Iser et al. [30]	2014	Cross-sectional	60,202	–	>18	Five regions: North, Northeast, Midwest, Southeast, South	Personally
Longo et al. [31]	2007	Cross-sectional	2022	61.5	20–59	Lages, SC	Personally
Machado et al. [32]	2005	Cross-sectional	377	100.0	45–64	Belo Horizonte, MG	Personally
Machado et al. [33]	2011	Cross-sectional	622	100.0	>50	Campinas, SP	Personally
Monteiro et al. [34]	2003	Cross-sectional	2122	59.7	>18	São Paulo, SP	By phone
Moreira et al. [35]	1991	Cohort	982	55.7	>18	Porto Alegre, RS	Personally
Munhoz et al. [36]	2012	Cross-sectional	2925	58.9	>20	Pelotas, RS	Personally
Peixoto et al. [37]	2005	Cross-sectional	2002	62.4	>18	Goiânia, GO	By phone
Pinheiro et al. [38]	2006	Cross-sectional	2420	70.0	>40	Five regions: North, Northeast, Midwest, Southeast, South	Personally
Schellini et al. [39]	2007	Cross-sectional	4690	63.6	>30	São Paulo	Personally
Schmidt et al. [40]	2006	Cross-sectional	54,369	60.8	>18	Five regions: North, Northeast, Midwest, Southeast, South	By phone
Theme-Filha et al. [41]	2003	Cross-sectional	5000	–	>18	Five regions: North, Northeast, Midwest, Southeast, South	Personally
Vianna et al. [42]	2010	Cross-sectional	2112	57.1	>20	Pelotas, RS	Personally
VIGITEL [43]	2007	Cross-sectional	54,251	60.3	>18	26 Brazilian capitals and Distrito Federal	By phone
VIGITEL [44]	2008	Cross-sectional	54,353	60.6	>18	26 Brazilian capitals and Distrito Federal	By phone
VIGITEL [45]	2009	Cross-sectional	54,367	60.7	>18	26 Brazilian capitals and Distrito Federal	By phone
VIGITEL [46]	2010	Cross-sectional	54,339	61.8	>18	26 Brazilian capitals and Distrito Federal	By phone
VIGITEL [47]	2011	Cross-sectional	54,144	58.2	>18	26 Brazilian capitals and Distrito Federal	By phone
VIGITEL [48]	2012	Cross-sectional	45,448	61.7	>18	26 Brazilian capitals and Distrito Federal	By phone
VIGITEL [49]	2013	Cross-sectional	52,929	61.7	>18	26 Brazilian capitals and Distrito Federal	By phone
VIGITEL [50]	2014	Cross-sectional	40,853	62.0	>18	26 Brazilian capitals and Distrito Federal	By phone
Fasting glucose diagnosis							
Cipullo et al. [51]	2005	Cross-sectional	1717	51.2	>18	São José do Rio Preto, SP	Fasting capillary glucose
Makdisse et al. [52]	2004	Cross-sectional	1159	53.3	>18	Five regions: North, Northeast, Midwest, Southeast, South	Fasting capillary glucose
Nunes Filho et al. [53]	2006	Cross-sectional	353	50.7	20–59	Luzerna, SC	Fasting glucose
Passos et al. [54]	1997	Cross-sectional	2310	59.0	>18	Bambuí, MG	Fasting glucose
Rodrigues et al. [55]	2000	Cross-sectional	1346	52.1	25–64	Vitória, ES	Fasting capillary glucose
Schaan et al. [56]	2000	Cross-sectional	992	52.6	>20	Rio Grande do Sul	Fasting glucose
Souza et al. [57]	2001	Cross-sectional	1039	51.0	>18	Campos dos Goytacazes, RJ	Fasting glucose
Complex diagnosis							
Bosi et al. [58]	2008	Cross-sectional	1116	64.5	30–79	São Carlos, SP	Capillary glycemia <200 mg/dL → OGTT
Malerbi et al. [59]	1988	Cross-sectional	21,847	59.0	30–69	Five regions: North, Northeast, Midwest, Southeast, South	Capillary glycemia <200 mg/dL → OGTT
Moraes et al. [60]	2007	Cross-sectional	2182	–	>30	Ribeirão Preto, SP	Capillary glycemia <200 mg/dL → OGTT
Oliveira et al. [61]	1989	Cross-sectional	2051	57.2	30–69	Rio de Janeiro, RJ	Capillary glycemia → OGTT
Rodrigues Júnior. et al. [62]	2011	Cross-sectional	1429	57.9	30–69	Campo Grande, MS	Capillary glycemia <200 mg/dL → OGTT OGTT >200 mg/dL → diabetes Capillary glycemia >200 mg/dL → diabetes
Schmidt et al. [63]	2010	Cohort	15,105	54.4	35–74	Northeast, Southeast, South.	Self-report or fasting glycemia ≥126 mg/dL or OGTT ≥200 mg/dL or HbA1c ≥6.5 %
Torquato et al. [64]	1997	Cross-sectional	1473	66.5	30–69	Ribeirão Preto, SP	Capillary glycemia → OGTT

**Fig. 2:**
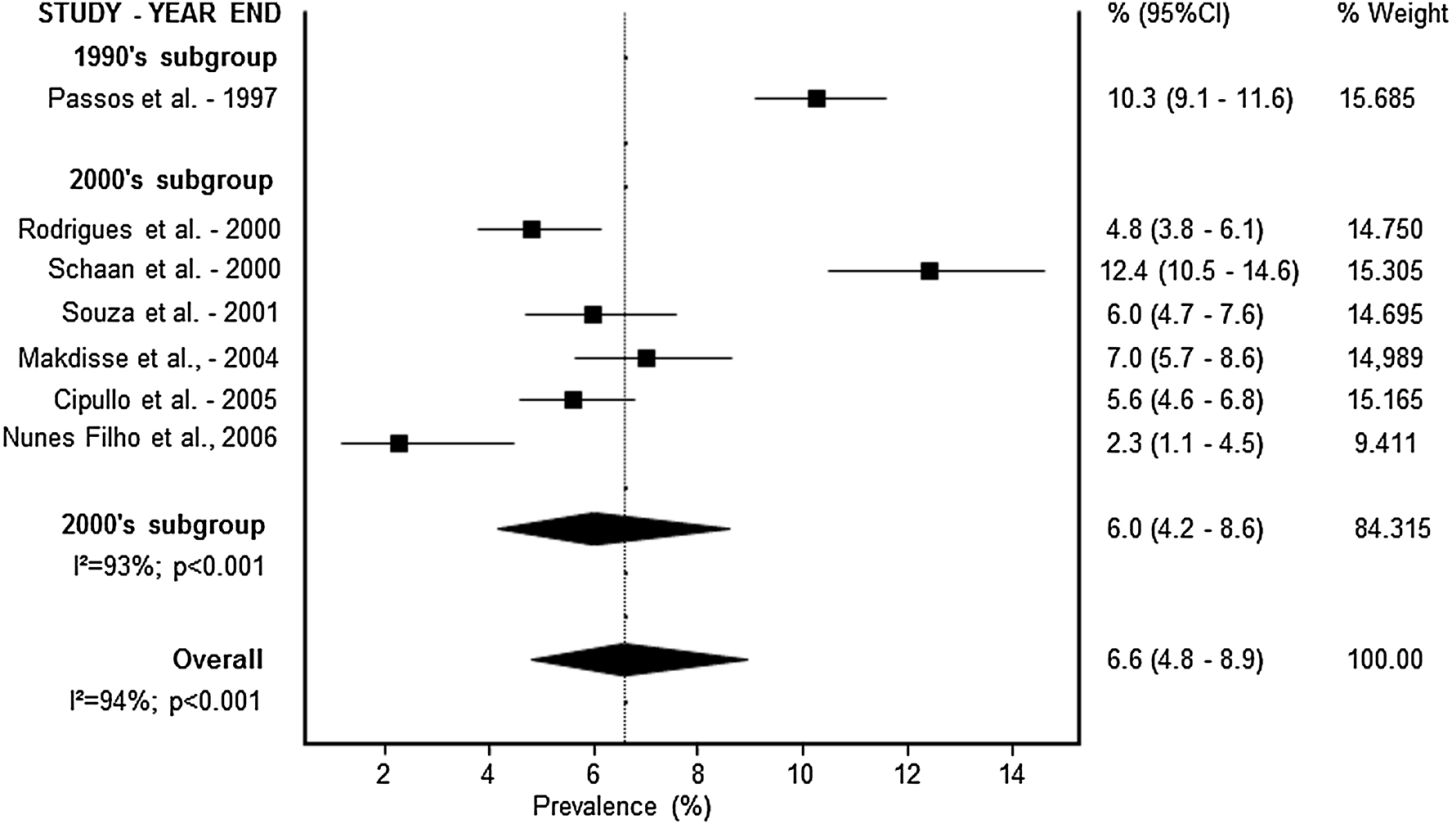
Forest plot representing diabetes prevalence rates by fasting glucose diagnosis and decades

**Fig. 3:**
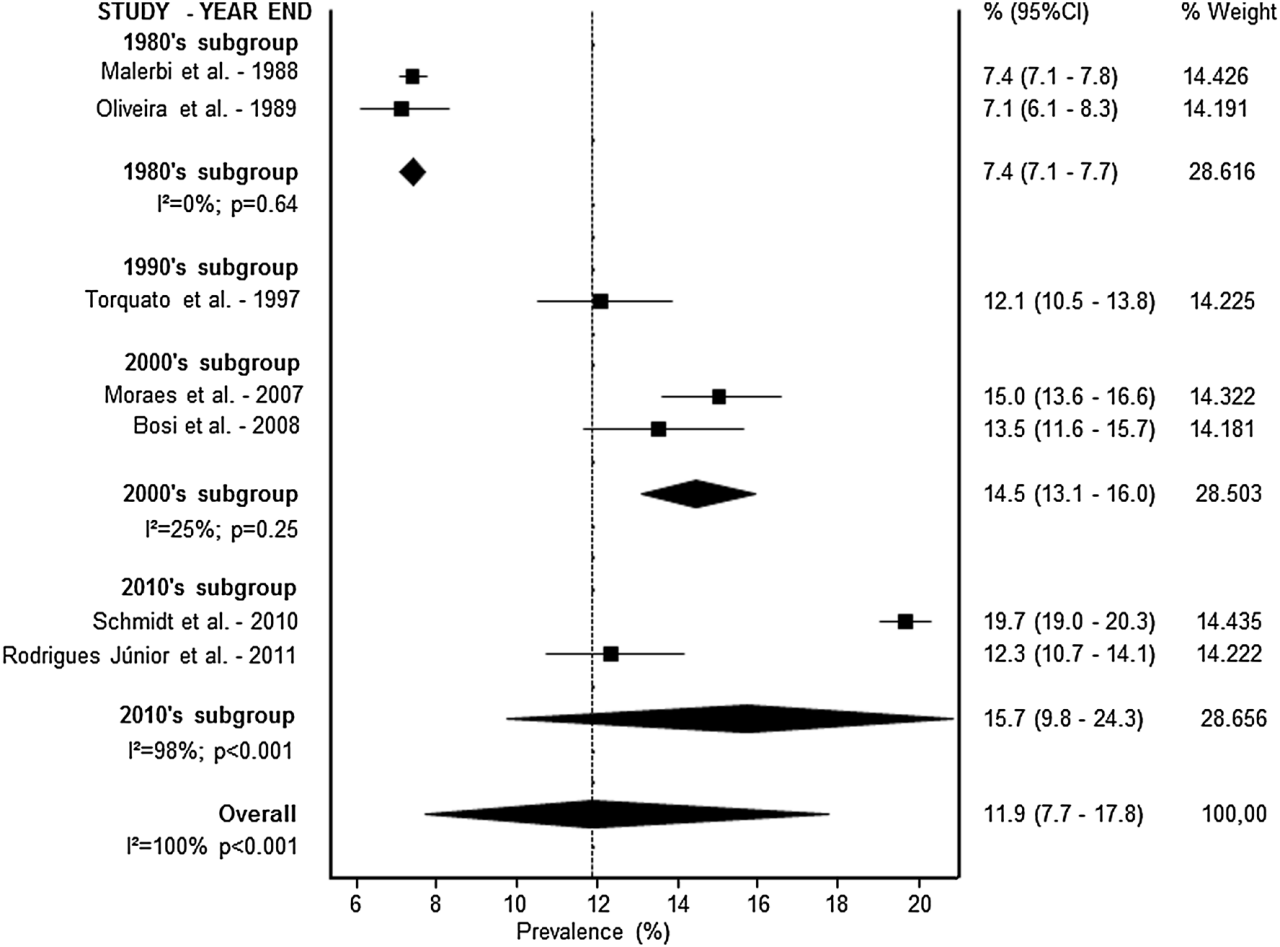
Forest plot representing diabetes prevalence rates by complex diagnosis and decades

A meta-analysis was conducted according to the diagnosis pattern. Prevalence rates are presented in Figs. 1 (self-reported), 2 (fasting glucose) and 3 (complex diagnosis), respectively. The prevalence of diabetes was 5.6 % (95 % CI 5.0–6.3) by self-report, 6.6 % (95 % CI 4.8–8.9) by fasting glucose, and 11.9 % (95 % CI 7.7–17.8) by complex diagnosis. In trend analyses, we observed an increase in the prevalence of diabetes in studies using a self-reported diagnosis [3.2 % (95 % CI 2.6–4.1) in the 1990s, 5.7 % (95 % CI 5.1–6.4) in the 2000s, and 6.9 % (95 % CI 6.2–7.6) in the 2010s] and studies using a complex diagnosis [7.4 % (95 % CI 7.1–7.7) in the 1980s, 12.1 % (95 % CI 10.5–13.8) in the 1990s, 14.5 % (95 % CI 13.1–16.0) in the 2000s, and 15.7 % (95 % CI 9.8–24.3) in the 2010s]. Only one study evaluated the prevalence of diabetes by fasting glucose in the 1990s (10.3 %; 95 % CI 9.1–11.6); the other six studies were conducted in the 2000s (6.0 %; 95 % CI 4.2–8.6). High statistical heterogeneity was identified in all analyses (data presented in Figs. 1, 2, 3).

In comparison to males, a female preponderance with regard to prevalence of diabetes was seen in this study in all decades and diagnosis criteria. Trends for the last three decades of diabetes prevalence by sex and decades are presented in Additional file 3: Figure S2 (self-reported), Additional file 4: Figure S3 (fasting glucose) and Additional file 5: Figure S4 (complex diagnosis). Prevalence rates of diabetes and their 95 % CI by sex, age group, regions, and adjustment to the design effect are presented in Table 2. The data presented in this table shows that the prevalence of diabetes was similar among the five different Brazilian macro-regions, and higher in older people. When analyses were adjusted to the design effect, the prevalence of diabetes was lower than the prevalence observed in not adjusted analyses.

**Table 2:** Subgroup meta-analysis of diabetes prevalence in Brazilian adults by diagnosis type and sex

Variables	Overall			Female			Male		
	N	% (95 % CI)	*I* ^2^ %	N	% (95 % CI)	*I* ^2^ %	N	% (95 % CI)	*I* ^2^ %
Self-reported									
Age group (years)									
20–69	5	5.1 (2.6–9.8)	99	4	5.1 (3.3–7.8)	98	2	2.7 (0.7–9.1)	99
>18	23	5.5 (4.8–6.3)	100	21	6.1 (5.5–6.9)	99	20	5.0 (4.2–6.0)	99
>30	3	7.2 (4.8–10.6)	97	4	9.0 (4.8–16.3)	98	3	6.0 (3.7–9.7)	92
Region									
South	8	6.2 (5.4–7.1)	78	5	7.3 (6.9–7.7)	0	3	5.5 (4.8–6.4)	0
Southeast	6	5.0 (2.7–8.9)	99	7	7.4 (4.7–11.6)	97	6	3.9 (1.9–8.0)	99
Midwest	1	4.4 (3.6–5.4)	–	1	4.4 (3.4–5.7)	–	1	4.3 (3.1–6.0)	–
Northeast	1	5.1 (3.8–6.9)	–	1	4.7 (4.2–5.3)	–	–	–	–
Multiples/national	15	5.7 (4.9–6.8)	100	15	5.8 (5.0–6.7)	100	15	5.2 (4.2–6.3)	100
Adjusted^a^									
Yes	15	5.3 (4.5–6.2)	99	16	5.7 (4.9–6.5)	99	16	4.5 (3.7–5.4)	99
No	16	5.9 (4.4–7.9)	99	13	7.4 (5.4–9.9)	99	9	5.6 (3.2–9.5)	99
Overall	*31*	5.7 (5.1–6.4)	100	29	6.3 (5.7–7.1)	100	25	4.9 (4.1–5.7)	99
Fasting glucose									
Age group (years)									
20–69	2	3.6 (1.7–7.3)	76	2	2.0 (0.3–12.9)	76	2	5.1 (3.8–6.8)	0
>18	5	6.0 (4.7–7.6)	93	3	9.5 (6.5–13.7)	8.7	3	9.0 (6.3–12.7)	83
Region									
South	2	5.6 (1.0–25.8)	96	2	3.2 (0.1–42.9)	90	2	7.6 (2.4–21.4)	89
Southeast	4	6.4 (4.3–9.5)	94	3	6.7 (3.4–12.5)	94	3	6.8 (4.9–9.3)	73
Multiples/national	1	7.0 (5.7–8.6)	–	–	–	–	–	–	–
Adjusted^a^									
Yes	1	6.0 (4.7–7.6)	–	1	5.7 (4.0–8.0)	–	1	6.3 (4.5–8.8)	–
No	6	6.7 (4.7–9.4)	94	4	7.2 (4.1–12.3)	92	4	7.5 (4.9–11.4)	86
Overall	7	6.6 (4.8–8.9)	94	5	6.8 (4.2–11.0)	92	5	7.3 (5.1–10.3)	84
Complex diagnosis									
Age group (years)									
30–69	4	9.4 (7.0–12.6)	96		9.6 (7.2–12.5)	93	4	9.0 (6.4–12.4)	92
Others^b^	3	16.0 (12.5–20.4)	96		16.3 (15.7–17.1)	0		16.6 (11.1–24.1)	95
Region									
Southeast	4	11.6 (8.4–15.7)	95	4	12.8 (8.9–17.2)	94	4	10.7 (6.7–16.7)	93
Midwest	1	12.3 (10.7–14.1)	–	1	12.1 (10.0–14.4)	–	1	12.6 (10.2–15.5)	–
Multiples/national	2	12.3 (4.5–29.5)	100	2	11.4 (3.4–21.2)	100	2	13.3 (4.1–35.8)	100
Adjusted^a^									
Yes	6	11.5 (6.4–17.7)	100	6	11.5 (7.5–16.2)	99	6	11.0 (5.9–19.5)	99
No	1	15.0 (13.6–16.6)	–	1	17.0 (15.2–18.9)	–	1	16.6 (13.9–19.6)	–
Overall	7	11.9 (7.7–17.8)	100	7	12.2 (8.4–16.6)	99	*7*	11.7 (6.8–19.3)	99

Complex diagnosis: OGTT + fasting glucose + self-reported, e.g

^a^Adjustment to the design effect

^b^Studies (age): Bosi et al. [58] (30–79); Moraes et al. [60] (>30) and Schmidt et al. [63] (35–74)

### Quality of studies

Additional file 6: Figure S5 summarizes data regarding quality of studies. Most studies were based on cross-sectional design (49 studies, 96 %). Three different patterns of diabetes diagnosis were identified and included in this analysis: self-report (36 studies, 72 %), fasting glucose (7 studies, 14 %), and complex diagnosis (7 studies, 14 %). Sample sizes varied substantially with a mean of 27.521 people. The mostly used design was cluster sampling (45 studies, 90 %), and sample size calculation was well described in 42 studies (84 %). Most studies were developed only in or including data from Southeast and Southern Brazil (35 studies, 70 % and 30 studies, 60 %, respectively), and 25 studies (50 %) included data from rural areas. Most studies did not have selection bias with potential to compromise internal validity (32 studies, 64 %), as well as analysis bias (43 studies, 86 %); however, only ten studies (20 %) appropriately described handling of missing data.

## Discussion

Decades ago, the global epidemic of diabetes was predicted by epidemiologists who observed large and rapid increases in the prevalence of type 2 diabetes related to Western lifestyle [65]. Over time, the burden of diabetes has taken place in developing rather than in developed countries [66]. In the present systematic review with meta-analysis of cross-sectional and baseline of cohort studies, which included more than one million individuals, it was possible to estimate the prevalence of diabetes in Brazil by decades, sex, macro-region, diagnosis criteria, and methods of assessment. Studies based on a complex diagnosis showed a high prevalence of diabetes in Brazilian adults (11.9 %), with a progressive increase in the last 35 years. This trend was also apparently observed in studies based on self-reported diagnosis.

The prevalence of diabetes in Brazil by self-reported method was lower than the other methods used in this study (complex diagnosis and fasting glucose); however, it was observed a progressive increase in prevalence for all detection methods in the last 35 years. This finding may be, in part, due to greater access to diagnostic testing [67], as well as the recognition of different diagnostic criteria tests for diabetes diagnosis [68]. A same telephone survey platform, which yearly evaluates diabetes prevalence in Brazil, identified an increase in the overall prevalence of diabetes from 6.3 to 8 % in just 5 years, a 21 % jump [46, 50].

Moreover, the diagnosis criteria for diabetes based on OGTT and fasting glucose became broader over time, which may differentially identify people without previous diagnosis as having diabetes [69]. It may explain, to some extent, the progressive increase in diabetes prevalence, as different biomarkers and definitions for diabetes may provide different estimates of population prevalence. Different biomarkers have been used to define diabetes, including fasting glucose, OGTT, and, more recently, HbA1c [69]. In this meta-analysis, only one study included HbA1c as diagnosis criteria for diabetes [40]. Results showed that approximately 10 % of cases were diagnosed only by HbA1c method, which can help to explain the high prevalence found. In this study, the prevalence of diabetes remained similar after adjustment by gender, age, skin color, weight status, and education level [40]. Population-based health surveys in different countries and at different times have also used different biomarkers and criteria for diabetes diagnosis, which define diabetes differently. This variety of biomarkers and definitions creates a challenge in consistently analyzing diabetes prevalence over time. In a large international pooled analysis of population-based examination surveys, it was found that the use of these different biomarkers and definitions can lead to different estimates of population prevalence of diabetes, with the highest prevalence observed when diabetes was defined based on fasting glucose or OGTT [69].

Although changing definitions could result in variations in diabetes prevalence over time, it doesn’t underlie the global increase in diabetes. As obesity is the most important risk factor for type 2 diabetes, its observed increase also influences the current trend in diabetes prevalence. Some studies suggest that more than 80 % of cases of type 2 diabetes can be attributed to obesity, which may also account for many diabetes-related deaths [70, 71]. The increases in the prevalence of type 2 diabetes, especially in older adults, may also reflect the reduction in major complications and mortality related to diabetes. Between 1988 and 2010, the largest increase in diabetes prevalence in the United States was observed in older adults (≥65 years), and only in this age group this increase remained significant after adjustment for body mass index or waist circumference [72]. Furthermore, the incidence of diabetes has been stable in the last years [73]. At the same time, the rates of the major diabetes-related complications, especially acute myocardial infarction, have declined [74]. In Brazil, the mortality related to diabetes decreased from 1996 to 2011, likely due to better and earlier treatment of the disease, given that the prevalence of diabetes continues to increase. This reduction was approximately twice larger in women (30 vs. 14 %) over 15 years, suggesting a possible role in the higher prevalence observed for this gender [3]. Possible reasons for the declines in mortality in Brazil over this period include the expanded public health system, especially in terms of primary care, with national programs focused on diabetes [75, 76], organization of emergency care facilities, and hot line systems for diabetes support [75].

In this study, we identified a pronounced female preponderance in diabetes prevalence in all decades, using different diagnosis criteria and methods of assessment. This finding is consistent with those observed in other studies, in which sex-related differences in genetics and lifestyle may lead to differences in the risk of developing diabetes and, in consequence, differences in the prevalence of this condition by sex [77]. In Brazil, a comprehensive literature review summarized the prevalence of diabetes through nine studies [9]. In this study, women were more likely than men to report having diabetes. Although this could be easily explained by the fact that higher incidence of diabetes is related to more frequently reported prevalence of diabetes, this finding may also reflect higher use of medical care by women and therefore increased likelihood of being diagnosed. However, this is not a consensus in the literature. Although previous studies in Brazil have found similar results [63], according to the Centers for Disease Control and Prevention, from 1980 to 1998, the age-adjusted diabetes prevalence by sex was similar in the United States, and, from 1999 on, the rate for males began to increase at a faster rate than that for females [78]. More studies controlling for lifestyle differences may help to better understand these findings.

The present study has some limitations. First, the lack of adjustment of some studies for design effect may compromise accuracy of estimate confidence intervals. Moreover, the different assessment methods for diabetes, changes in diagnosis criteria over time, and high heterogeneity found among the studies limit the interpretation of our results. Also, the poor coverage of the evidence base in many Brazilian regions restricts evaluation for obtaining the national diabetes prevalence over time. Our projections for diabetes prevalence trends for the last three decades are relied on demographic statistics, which might not be accurate for many regions and populations. As a result, our findings should not be considered an appropriate equivalent of a nationwide prevalence study. Also, specific therapies and better social and medical care may reduce direct and indirectly associated mortality and increase prevalence. Further studies in all five Brazilian macro-regions should be required to take into account potential racial, cultural, and socioeconomic diversity of this nation as a whole.

In conclusion, despite the high heterogeneity, this systematic review with meta-analysis showed a high prevalence of diabetes in Brazilian adults over time, with a progressive increase in the last 35 years. These findings may be, in part, associated with improvement in access to health services in the same period. Nevertheless, this study has important epidemiological implications. Our findings reinforce the significant rise in the prevalence of diabetes, which may result in heavy health burden associated to this disorder and its related complications. Based on our results, further studies are necessary to better understand the factors associated to the increasing diabetes prevalence in Brazil, which may help to shape future prevention programs.

## Additional files

10.1186/s13098-016-0181-1 Literature search strategy used for the pubmed database.
10.1186/s13098-016-0181-1 Flow diagram: identification and selection of articles included in the meta-analysis.
10.1186/s13098-016-0181-1 Forest plot representing diabetes prevalence rates by self-report and decades in (A) women and (B) men.
10.1186/s13098-016-0181-1 Forest plot representing diabetes prevalence rates by fasting glucose and decades in (A) women and (B) men.
10.1186/s13098-016-0181-1 Forest plot representing diabetes prevalence rates by complex diagnosis and decades in (A) women and (B) men.
10.1186/s13098-016-0181-1 Quality of studies characteristics.
