# Supplementary material for: Prevalence of diabetes in Brazil over time: a systematic review with meta-analysis
Source: Diabetol Metab Syndr. 2016 Sep 7;8(1):65. doi: 10.1186/s13098-016-0181-1 (PMC5015260; doi:10.1186/s13098-016-0181-1)
Supplement: Supplementary file 1 — 10.1186/s13098-016-0181-1 Literature search strategy used for the pubmed database. [file 13098_2016_181_MOESM1_ESM.pdf]

Table S1. Literature Search Strategy Used for the PubMed Database

#1 Search: "Diabetes Mellitus, Type 2"[Mesh] OR "Diabetes Mellitus, Type 2" OR “Type 2 Diabetes” [title/abstract] OR “Diabetes Mellitus Type II” [title/abstract] OR “Diabetes Mellitus” [Mesh] OR “Diabetes Mellitus” [Title/Abstract] OR “Blood glucose” [Mesh] OR “Blood glucose” [Title/Abstract] OR “Hemoglobin A, Glycosylated” [MesH] OR “Hemoglobin A, Glycosylated” [title/abstract] OR Hba1c [title/abstract] OR “Ketosis-Resistant diabetes” [title/abstract] OR “Ketosis Resistant diabetes” [title/abstract] OR “Maturity-Onset diabetes” [title/abstract] OR “Non Insulin Dependent diabetes” [title/abstract] OR “stable Diabetes” [title/abstract] OR “Maturity Onset Diabetes Mellitus” [title/abstract] OR MODY [title/abstract] OR NIDDM [title/abstract] OR “Adult-Onset Diabetes Mellitus” [title/abstract] OR “Diabetes Mellitus Noninsulin Dependent” [title/abstract] OR “Diabetes mellitus/epidemiology”[Majr] OR “Diabetes mellitus/statistics and numerical data”[Majr]

#2 Search: Brazil [Mesh] OR Brazil* [Title/Abstract] OR Brazil [Title/Abstract] OR “Minas Gerais” [Title/Abstract] OR “São Paulo” [Title/Abstract] OR “Espirito Santo” [Title/Abstract] OR “Rio de Janeiro” [Title/Abstract] OR Bahia [Title/Abstract] OR Pará [Title/Abstract] OR “Mato Grosso” [Title/Abstract] OR “Mato Grosso do Sul” [Title/Abstract] OR Goiás [Title/Abstract] OR “Rio Grande do Sul” [Title/Abstract] OR Ceará [Title/Abstract] OR Pernanbuco [Title/Abstract] OR “Santa Catarina” [Title/Abstract] OR Amazonas [Title/Abstract] OR Maranhão [Title/Abstract] OR Tocantins [Title/Abstract] OR Piauí [Title/Abstract] OR Rondônia [Title/Abstract] OR Roraima [Title/Abstract] OR Paraná [Title/Abstract] OR Acre [Title/Abstract] OR Amapá [Title/Abstract] OR Paraíba [Title/Abstract] OR “Rio Grande do Norte” [Title/Abstract] OR Alagoas [Title/Abstract] OR Sergipe [Title/Abstract] OR “Distrito Federal” [Title/Abstract]

#3 Search: Prevalence [Mesh] OR Prevalence [Title/Abstract]

#4 Search: #1 AND #2 AND #3
