# Supplementary figures and images for: Prevalence of diabetes in Brazil over time: a systematic review with meta-analysis
Source: Diabetol Metab Syndr. 2016 Sep 7;8(1):65. doi: 10.1186/s13098-016-0181-1 (PMC5015260; doi:10.1186/s13098-016-0181-1)

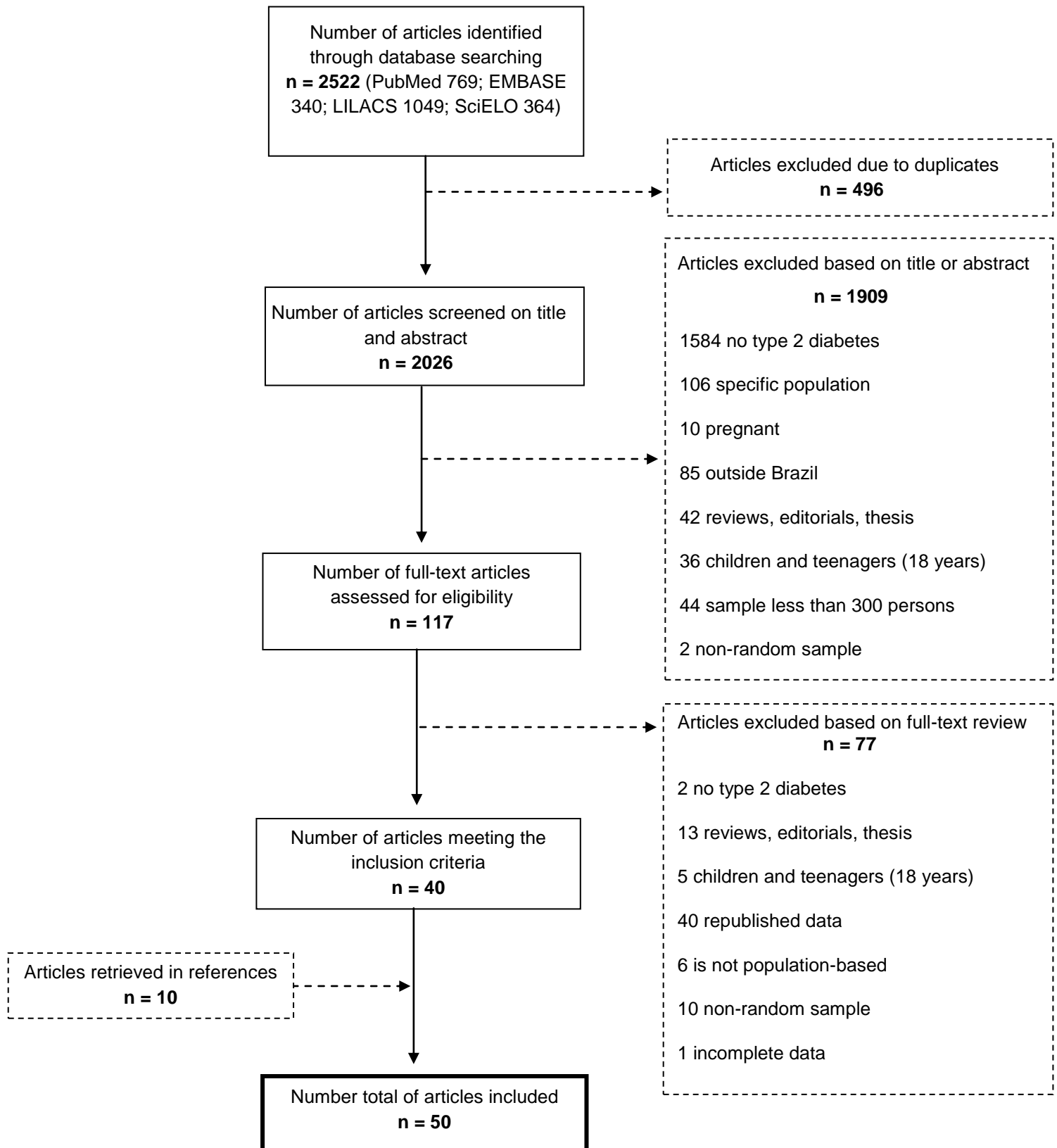

**Figure S1.** Flow diagram: identification and selection of articles included in the meta-analysis

Supplement: Supplementary file 2 — 10.1186/s13098-016-0181-1 Flow diagram: identification and selection of articles included in the meta-analysis. [file 13098_2016_181_MOESM2_ESM.pdf]

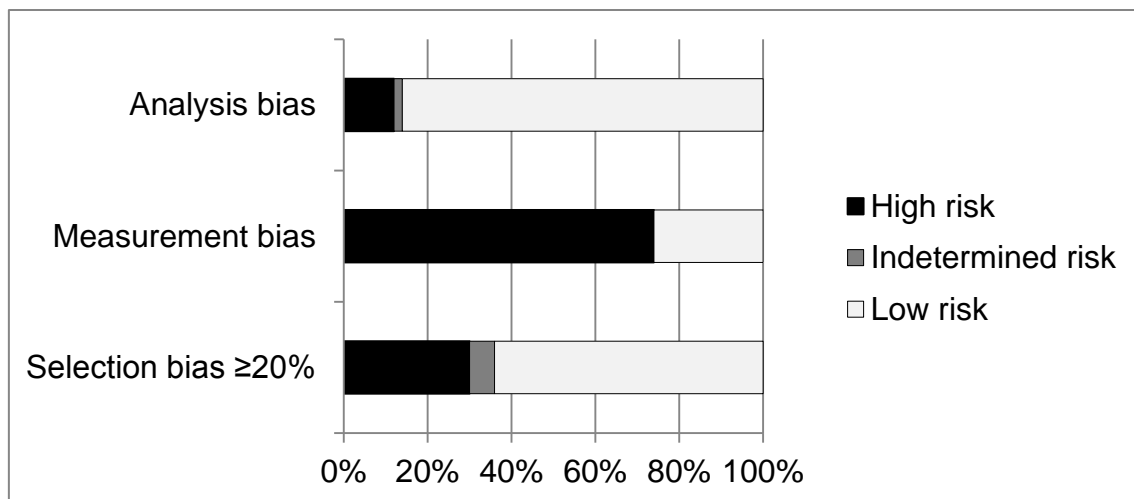

**Figure S5.** Quality of studies characteristics

Supplement: Supplementary file 6 — 10.1186/s13098-016-0181-1 Quality of studies characteristics. [file 13098_2016_181_MOESM6_ESM.pdf]
