# Supplementary material for: Prevalence of diabetes in Brazil over time: a systematic review with meta-analysis
Source: Diabetol Metab Syndr. 2016 Sep 7;8(1):65. doi: 10.1186/s13098-016-0181-1 (PMC5015260; doi:10.1186/s13098-016-0181-1)
Supplement: Supplementary file 4 — 10.1186/s13098-016-0181-1 Forest plot representing diabetes prevalence rates by fasting glucose and decades in (A) women and (B) men. [file 13098_2016_181_MOESM4_ESM.pdf]

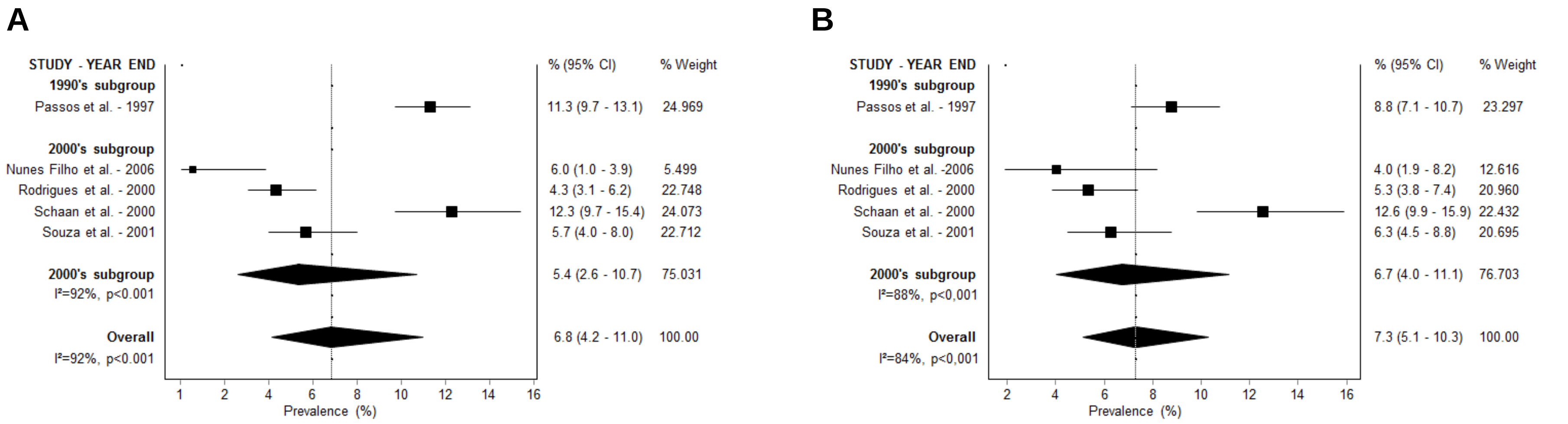

Figure S3. Forest plot representing diabetes prevalence rates by fasting glucose diagnosis and decades in (A) women and (B) men
